# The Temperance Warfare

**Published:** 1874-10

**Authors:** 


### THE TEMPERANCE WARFARE.

There is not a nation on earth, nor has there ever been, civilized or savage, which has not discovered some form of artificial alcoholic stimulant. Wine was known beyond the flood. Drunkenness will abound, with all its disastrous consequences, until the Milennium. An incalculable amount of good has been done by the friends and advocates of temperance in curtailing the evil, for which all the good can never be too thankful. But there are two errors which the friends of the great reform have committed, and error can never build up truth. No truth can be sustained by false principles. These errors are one of principle and one of practice. The friends of temperance have contended for too much. They have not yet adopted the true plan of remedying the evil. As evidence of this last, the mode of attack has been constantly changing since the organization of the original temperance society. First whiskey was attacked; then wine; then cider; then everything which could intoxicate; then what could not intoxicate, as beer, ale and porter. Still, intemperance flowed as a river, devastating all in its course, more terrific than the Massachusetts and the Pittsburgh floods. Next legislative interference was invoked; later on, the prayers of women in the public streets; and if the friends of temperance were numerous and powerful enough, they would employ a despotism equal to that of any autocrasy on the globe. The irresistible tendency of the age is to liberty of thought and action, personal and mental moral liberty; and this will go on increasing until the end of time. Whatever cannot be done as to the masses in civilized society by

MORAL SUASION

will never be done at all. That is the Almighty's method of peopling heaven; not by the thunder and lightning and storm and tempest and earthquake, but by the "still, small voice" of reason, through spiritual influences on the heart. The utmost that can be hoped for in this world is the temperate use of stimulants; to keep the people within the bounds of this temperate use, they must first be persuaded to become christians, who will "use these things as not abusing them." The friends of temperance have contended for too much in advocating total abstinence from everything that can intoxicate. True, it is the surest and the only safe way; at the same time, however, society, constituted as it is, will never submit to the prohibition. Another great error is in the tendency to outlaw everybody that ever tastes liquor; many not hesitating to say, that no man can be a christian who is not in principle and practice a total abstinent; this is an intolerable intolerance.

Learned theologians have done an incalculable injury to the Bible, and to our common christianity, in attempting to prove that the wine of the scriptures did not intoxicate; that there was a wine in common use which did not intoxicate. The Bible does not teach total abstinence from all that intoxicates, but it does teach the temperate use of stimulants; temperance in all things, in the use of wine, but no more decidedly than the temperate indulgence of the animal appetites and passions. It is not the plan of the Beneficent One to bind his creatures in chains, to keep them from hurting themselves. A farmer will huddle up his pigs in a pen to keep them from devastating his corn. But men are not pigs; and they are turned into the field to eat and to destroy. Men are not driven into heaven; they are invited. They are persuaded to become christians; and if all the time and labor and money and thought which have been used to bring about total abstinence from things that can intoxicate had been expended in the direction of promoting christianity in the individual, there would have been less drunkenness than there is to-day, because it is the preaching of the gospel, the whole gospel, by man to man which is to make a heaven of earth; temperance in drink is but a very small part of that gospel, absolute abstinence is no part of it. Probably the wisest plan ever yet adopted to curtail the evils of intemperance is

LOCAL OPTION, which means that permission to sell liquor must be obtained by a majority of the legal voters of the place.

The use of stimulants should be allowed to the sick, to invalids, to weakly persons, and to old people, to all who are benefitted by that use who have reached the age of threescore years. If those who are young and vigorous, and those who are in good health, choose to use stimulants they will have to suffer for it, that's all; and that such will fall into beastly habits, and die prematurely and in disgrace is among the certainties.

After all, the safe side is the best; and they only are safe who never allow themselves to take a drop of liquor under any circumstances. Still, it is not a crime to do it. It is not a disgrace, not a moral poison, not an evidence of a candidacy for "the perdition of ungodly men," as

INTEMPERATE TEMPERATES

are constantly avowing, and to that extent they are hindering the great cause, bringing upon it the angry impatience of the ignorant and the contempt of all men of enlarged views.

